# Using Video Games to Improve the Sexual Health of Young People Aged 15 to 25 Years: Rapid Review

**DOI:** 10.2196/33207

**Published:** 2022-05-19

**Authors:** Ignacio Franco Vega, Anastasia Eleftheriou, Cynthia Graham

**Affiliations:** 1 Department of Social & Policy Sciences University of Bath Bath United Kingdom; 2 TRI Technologos Research and Innovation Services Ltd Larnaka Cyprus; 3 Department of Psychology Faculty of Environmental and Life Sciences University of Southampton Southampton United Kingdom

**Keywords:** sex education, serious games, sexually transmitted infections, rapid review, mobile phone

## Abstract

**Background:**

Sexually transmitted infections and unintended pregnancies among young people remain public health concerns in many countries. To date, interventions that address these concerns have had limited success. Serious games are increasingly being used as educational tools in health and professional public education. Although acknowledged as having great potential, few studies have evaluated the use of serious games in sexual health education among young people, and to date, there have been no published reviews of these studies.

**Objective:**

This study aims to assess the effects of video game–based sexual health interventions for risky sexual behavior in young people aged between 15 and 25 years.

**Methods:**

A rapid review of randomized controlled trials and quasi–randomized controlled trials was performed. The search included the following bibliographic databases: Cochrane Central Register of Controlled Trials, Embase, MEDLINE, PsycINFO, and Scopus. A total of 2 reviewers independently screened 50% (35/70) of the retrieved articles during the full-text screening phase.

**Results:**

From a total of 459 identified citations, after removing duplicates, 327 (71.2%) articles were deemed eligible for title and abstract screening. Of the 327 articles, 70 (21.4%) full texts were screened, from which 10 (3.1%) articles (evaluating 11 different games) were included in the review. The findings highlighted the considerable diversity in video game–based interventions and assessed sexual health outcomes. Although there were some promising findings in outcome studies using game-based interventions, the results across studies were mixed.

**Conclusions:**

Although game interventions for sexual health have been in existence for almost three decades, relatively few studies have evaluated them, and the results of previous outcome studies have been mixed. Moreover, there is little clarity regarding which specific elements of a game facilitate positive outcomes. We provide recommendations for future researchers developing video game–based interventions to improve sexual health in young people.

## Introduction

### Background

Although contraceptive access and sexual education are more widely available than ever, risky sexual behavior remains an issue for people of all ages, especially younger individuals. More than 1 million sexually transmitted infections (STIs) are acquired each day worldwide among individuals aged between 15 and 49 years [1,2]. The most common STIs are chlamydia, gonorrhea, and trichomoniasis, although the diagnosis of syphilis has also increased in recent years [1]. In many countries, young people aged between 15 and 24 years have the highest rates of STIs [3,4]. Despite global and national efforts to stop the spread of STIs, the World Health Organization recently reported a “concerning lack of progress” in achieving reductions [5].

The best method for preventing the spread of STIs is the correct use of condoms [6]. However, many young people [7] engage in risky sexual behaviors such as having sex with multiple partners without the use of condoms or incorrect or incomplete condom use [8]. Many interventions have been developed to encourage consistent condom use and safer sexual behaviors; however, most of these interventions have been shown to have limited effectiveness and/or are very resource intensive [9,10].

Adolescent pregnancies are also a global concern in high-, middle-, and low-income countries. Although the past 30 years have seen a global decline in unintended pregnancy rates, a recent review of 166 low- to middle-income countries reported that approximately half of all pregnancies are unintended [11]. Furthermore, although rates of unintended pregnancies in the United States and the United Kingdom have dropped in recent decades [12], adolescent pregnancy rates remain high in many middle- to high-income countries (particularly in the United States) [13].

Despite consistent evidence that comprehensive sex education can increase protective behaviors [14], there are still many gaps in knowledge. Furthermore, access to contraceptives and sexual health services for young people remains limited in many countries [4]. Sex education, often delivered in schools as part of the national curriculum, can be a highly contested area, reflecting political, moral, and cultural debates. In the United States, school-based sex education curricula have long been criticized for being sex negative, often focusing on abstinence and omitting any mention of nonheterosexual experiences [15,16]. In many countries, traditional gatekeepers such as religious and educational authorities still powerfully restrict access, content, and materials used for sex education [4]. Therefore, for many young people, obtaining reliable information about sex and relationships can be difficult.

The internet has been identified as a potentially valuable resource for comprehensive, interactive, web-based, and youth-friendly sex education [16]. Young people worldwide use the internet and social media to access information on sexual and reproductive health and rights [16,17]. Interventions delivered through digital media could particularly help reach marginalized groups such as young people in rural areas; lesbian, gay, bisexual, transgender, and intersex individuals; people with disabilities; and migrant populations [17].

It has been argued that education through games is more efficient and enjoyable than classroom teaching for several reasons [18]. First, it is predominantly the player who directs activity in games, whereas in school, it is predominantly the teacher who directs activity. This is why serious games use a learner-centered approach in which learners are involved in the process (learning through doing), in contrast to traditional education, which uses a teacher-centered approach in which learners are relatively passive.

Second, children and adolescents often find it difficult to properly engage in school exercises [19], in which the challenge level is not well adjusted to their skills. In a class, there are many students with different skills, making it difficult for teachers to equally engage all students in the class. In contrast, video games engage players naturally by gradually adjusting their difficulty level as they progress in the game [20]. Game developers understand that for a game to be successful, players of varying abilities need to feel a sense of reward or achievement, often enough to retain their engagement.

Third, students are sometimes discouraged by the school system as they are penalized for the mistakes they make (eg, they receive bad grades). However, in games, players are expected to make wrong decisions and do so without being discouraged (ideally, unless the game is poorly designed). In fact, games have the advantage of allowing users to train in real-life decision-making situations where the wrong choice may involve some risk without having to actually be at risk. For example, pilots often train using Microsoft Flight Simulator, whereas the military often uses battle simulators to train recruits. This allows players to make mistakes in a safe environment.

Finally, an important characteristic of educational games is the constant real-time feedback provided to the user. Players almost instantly know how well a certain move or strategy works toward the goal of the game. Feedback can take the form of points, lives, levels, scores, ranks, or progress bars. Real-time feedback ensures that users are motivated throughout the game by promising that a goal is achievable.

Some authors have argued that there is a strong case for integrating video games into sex education, whether by supplementing sex education classes with existing games that explore sex and sexuality or developing new games for the purpose of sex education [21]. Given the interactive nature of video games, their lack of real consequences, their capacity for privacy, and the familiarity that many adolescents already have with games, when used correctly, games could be very effective tools for students.

### Aims of the Review

This review was conducted as part of a larger Erasmus+ funded project (Safe4Play) that aims to develop an innovative tool for sex and reproductive health education for young people using serious games with machine learning features. The aim of this review was to analyze the core elements and effects of video game–based interventions for improving the sexual health of young people. The findings informed the development of the intervention that was produced as part of the Safe4Play initiative.

## Methods

We conducted a rapid review following the Cochrane Rapid Reviews Method Group guidelines [22,23]. A rapid review can be defined as a type of knowledge synthesis in which the usual procedures of a traditional systematic review are streamlined and accelerated such that the most crucial elements are still present, but the research time is considerably abridged [24].

### Criteria for Study Selection

The criteria for selecting studies were based on the Population, Intervention, Comparison, Outcomes, and Study characteristics framework.

#### Population

This involved interventions aimed at working with youth (aged 15-25 years). Where studies included participants who fell both inside and outside of our target bracket (eg, aged 12-16 years), we tried, where possible, to select the appropriate results from the subset of the sample that met our age criteria; if that was not possible, we captured that specific limitation in the narrative form.

#### Intervention

This involved any video game–based sexual health intervention aimed at reducing risky sexual behavior. We considered a *video game–based intervention* as an educational intervention delivered through an electronic or digital medium that relied heavily on game mechanics, aesthetics, or game thinking (competition, cooperation, exploration, and storytelling) to engage, motivate action, promote learning, and solve problems [25].

#### Comparison

This criterion was not applicable.

#### Outcomes

As we were broadly interested in sexual health, we chose to include studies that assessed a broad range of knowledge, attitudinal, and behavioral variables. We defined primary outcomes as any of the following: decrease in unintended pregnancies and STIs, increase in contraceptive use, increase in intention to use contraceptives, acquisition of new knowledge regarding sexual health, change in the perception of risk of pregnancy, and change in the perception of risk of STIs. Secondary outcomes included changes in attitudes toward safe sex, self-efficacy toward sexual health, decrease in the number of sexual partners, increase in safe and consensual relationship practices, and increase in adherence to pre-exposure prophylaxis (PrEP).

Where studies reported >1 relevant outcome, each one was captured and reported in a narrative form. When outcomes were provided at multiple follow-up points, all outcomes were reported for each follow-up point.

#### Study Characteristics

We included randomized controlled trials (RCTs) and quasi-RCTs (studies in which participants were allocated to different arms of the study using a method of allocation that is not truly random). Publications in either English or Spanish were considered.

### Search Strategy and Search Terms

The search strategy was validated by the Safe4Play research team and an information retrieval specialist from the University of Bath. It was piloted to analyze the quality and quantity of its results; only small changes were made based on the findings.

We used 5 databases to identify relevant studies: Cochrane Central Register of Controlled Trials, Embase, MEDLINE, PsycINFO, and Scopus. Searches were conducted on April 23, 2021. In addition, we hand-searched the reference lists of the included trials for referenced articles that were not retrieved in the original search. We also contacted experts in the field for additional recent publications that the original search might not have identified. For details of the search terms used for each of the databases, see Multimedia Appendix 1.

### Study Selection

A total of 2 steps were undertaken to assess the eligibility of the studies: title and abstract screening and full-text screening. A total of 2 reviewers (IFV and CG) were involved in the process. Approximately 20% (51/257) of the abstracts were independently screened by both reviewers, which served as a pilot to identify any salient issues. The remaining 80% (206/257) of the abstracts were screened by IFV. Interrater reliability was found to be moderate (weighted κ=0.53) [26]. All cases of uncertainty or discrepancy were resolved through discussions between the 2 reviewers.

In the full-text screening stage, both reviewers independently screened half of the articles to confirm whether the studies identified during the title and abstract screening should be included. Reliability was found to be substantial (κ=0.71) [26]. The same procedure was used to resolve any discrepancies between reviewers. The remaining articles were screened solely by IFV.

Data extraction was performed by IFV. All pertinent data were extracted from the full text using a spreadsheet template. When an intervention was analyzed in multiple papers, data from all papers were considered during the extraction.

## Results

### Search Results

**Figure 1:**
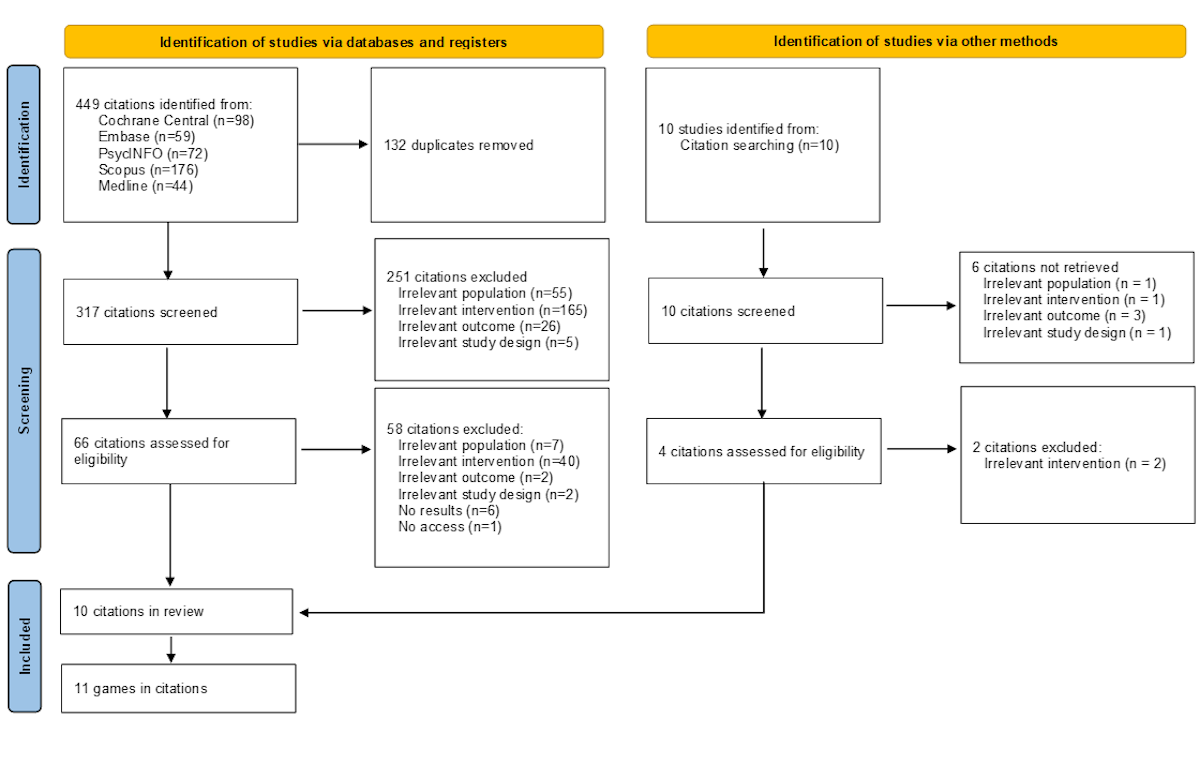
Flow diagram from the article selection process.

As shown in Figure 1, the search strategy produced 449 results, of which, after removing 132 (29.4%) duplicates, 317 (70.6%) articles remained (299/317, 94.3% of empirical papers, and 18/317, 5.7% of reviews). All systematic reviews were scanned to identify additional articles to screen; 10 additional articles were found through this process. A total of 327 abstracts were deemed appropriate for screening.

Overall, of the 327 articles found, 257 (78.6%) were screened at the title and abstract screening, leaving 70 (21.4%) articles for full-text screening. These 70 articles were downloaded and examined. After this final screening procedure of the 70 articles, 60 (86%) articles were excluded, leaving 10 (14%) articles with suitable games to analyze. Most articles described 1 game each, although one of the articles evaluated 2 games. Thus, the final search product was 11 games.

In some cases, to obtain the information required to conduct a proper analysis, additional supplementary materials had to be downloaded. Most of these were in the form of protocols for trials or articles that reported preliminary results. In the following sections, we briefly describe each of the identified games. Table 1 presents some of the key features (sample, location, and type of game) of each video game.

**Table 1:** Key features of included studies.

Game name	Target population	Age (years), range	Location and publication date	Console	Type of game
The Baby Game! [27]	High school students	13-18	Hawaii, United States, 1989	PC	Management simulator
Romance [27]	High school students	13-18	Hawaii, United States, 1989	PC	Text adventure
VODO [28]	High school students	15	New Orleans, United States, 1989	PC	Text adventure
Choose Your Own Adventure [29]	High school sophomores	15-16	Kentucky, United States, 2007	PC	Dating simulator
SOLVE^a^ [30]	Men who have sex with men	18-24	United States, 2013	PC	3D dating simulator
HIV risk game [31]	Youth	15-19	Cape Town, South Africa, 2015	PC	Quizzes
Keep it up! [32-34]	YMSM^b^	18-29	Atlanta, Chicago, and Georgia, United States, 2017	PC	Dating simulator and minigames
BattleViro [35,36]	Young patients of antiretroviral therapy	14-26	Mississippi, United States, 2018	Smartphone (only IOS^c^)	Twin-stick shooter and quizzes
Viral Combat [37]	YMSM	18-35	Mississippi, United States, 2021	Smartphone (only IOS)	Twin-stick shooter and quizzes
MyPEEPS [38-40]	Male youth sexually attracted to men	13-18	United States, 2019	PC and smartphone (any; usable as a web application)	Role-playing games
First-person scenario game^d^ [41,42]	College students	17-27	Hong Kong—China, 2020	Smartphone (nonspecified)	First-person dating simulator

^a^SOLVE: Socially Optimized Learning in Virtual Environments.

^b^YMSM: young men who have sex with men.

^c^IOS: iPhone Operating System.

^d^See Multimedia Appendix 2.

### Identified Games

#### The Baby Game!

*The Baby Game* [27] is a management simulator in which high school students are asked to simulate a budget and schedule based on different scenarios. The students’ task is to establish how many hours they could devote to different activities (eg, chores, homework, sleep, recreation, and caring for their baby). They receive feedback, printed on a scorecard, based on how close their schedules are to a hidden correct time distribution.

The game aims to provide realistic information about the life changes that would occur if a student had a baby and how the newly added responsibility might affect their lives, with the assumption that this would enhance young people’s intentions of delaying parenthood and using contraceptives.

#### Romance

*Romance* [27] is a text adventure in which high school students write down how they will deal with a set of scenarios of romantic and sexual nature. They then receive feedback in the form of a simulated outcome. At the end of their run, players obtain a final scorecard based on the adequacy of their decisions. The exercise aims to improve students’ knowledge about sexuality and contraception, increase their skills for interaction, and serve as a practice for responsible sexual decision-making.

#### VODO

*VODO* [28] is a text adventure game in which high school students aged 15 years have to guide the main character through a series of scenarios. The game presents the player with a detailed written description of a situation; for example, “You are in your room. It is a sunny room full of things that are important to you. Tell the computer what you want to do?” The players then respond using simple English sentences; the game has an extensive vocabulary and is able to anticipate the responses typically provided by the students. Efforts were made so that although the player needs to make many choices, decisions are not presented overtly. This was done because the researchers wanted to convey the lesson that one has a choice, even when apparent conditions suggest otherwise.

An important aspect of this game is that it includes a roster of nonplayer characters (NPCs) with whom a player can interact and even form relationships that may or may not involve sex. Each of the NPCs has different names, personalities, and motives. In cases where the player chooses to have unprotected sex, the game creates a scenario in which the character has a child. The child randomly cries for different reasons and requires careful attention, creating tension between the character and their friends.

*VODO* was designed to improve participants’ decision-making skills by providing a scenario in which they were able to rehearse and obtain feedback on their choices. The topics presented in the game were broad. Although they are focused on matters of sexual health (eg, contraceptive use, STIs, and the consequences of unwanted pregnancies), it also includes other issues that might affect teenagers (eg, drunk driving, drug use, and the ability to be alone without being lonely). Strategies such as complementary quizzes were meant to increase real-life communication about sex within the family.

#### Choose Your Own Adventure

*Choose your own adventure* [29] is the name that we have provided for 1 of the 6 modules that formed an unnamed intervention aimed at reducing rates of unintended pregnancy and STIs in adolescents from rural areas in the United States.

The game comprises half of one of the modules. Players are expected to play through a virtual date and make choices that could put them in a situation where their dates want to have sex, but they do not. The game finishes with different positive or negative outcomes and products of the in-game decisions that were taken. To make the game more engaging and increase its replay value, the developers built in some remarkable elements. For example, they included >150 images of various people, places, and STIs, which were randomly selected at various points in the game so that they would be different during each run. Furthermore, all in-game dialogs were recorded, and the NPCs actually spoke to the players. The other half of the module comprised submitting an original refusal line. The researchers reported that the entire module (game+refusal line submission) had a completion rate of 41%.

#### Socially Optimized Learning in Virtual Environments

**Figure 2:**
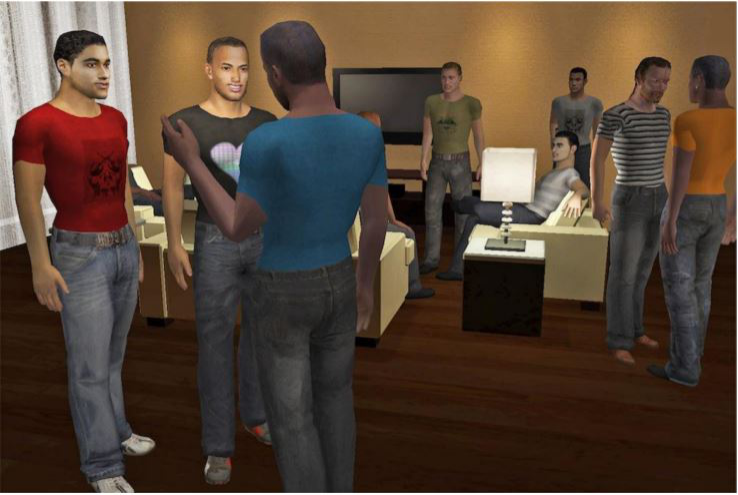
SOLVE (Socially Optimized Learning in Virtual Environments) avatars [30].

*SOLVE* (Socially Optimized Learning in Virtual Environments) [30] is a 3D dating simulator aimed at men who have sex with men (MSM) aged 18 to 24 years who reported having engaged in recent unprotected anal intercourse (UAI). The settings are constructed around different scenarios that might be faced by young MSM involving some form of sexual decision (eg, meeting someone at a party and going to their apartment afterward). In each situation, the player encounters a series of *choice points* where they need to make self-regulatory decisions (eg, accepting or refusing alcohol or offers of casual sex). After choosing to engage (or not) in virtual sex, there is a customized recap sequence in which the player’s virtual behavior is shown in sequence so that he can identify the different decisions that led to a particular outcome (Figure 2).

The idea was that through rehearsal and feedback, players could practice their decision-making skills. Throughout the process, they are guided by different NPCs (peers and one’s virtual future self) who instruct them to follow a set of guidelines when faced with risky situations.

#### HIV Risk Game

This intervention [31], unnamed in the published article, is a relatively simple game in which youth are expected to identify who was more likely to have HIV between 2 randomly generated individuals. Each participant plays 10 rounds of the game. Instead of receiving a direct answer regarding whether they made the right choice, the participants receive information about HIV and risk and construct their own learning based on their experimentation.

#### Keep it Up!

*Keep it up!* [32-34] is a 7-module, multi-method intervention (one that includes the use of >1 method of data collection in a study) aimed at improving STI prevention strategies among young MSM in the United States. The main gaming component is called The Club Game. This game uses a real-life scenario (going to the club) to explore decision-making around using condoms; the steps to use condoms properly; and the effects of excessive alcohol consumption, drug use, and sexual arousal on decision-making. The player goes through 5 rooms and interacts with other patrons while completing the activities related to the abovementioned topics. The intervention uses diverse delivery methods (eg, videos, animation, and games) to improve HIV knowledge, motivate safer behaviors, teach skills, and increase self-efficacy for preventive behaviors.

#### BattleViro

**Figure 3:**
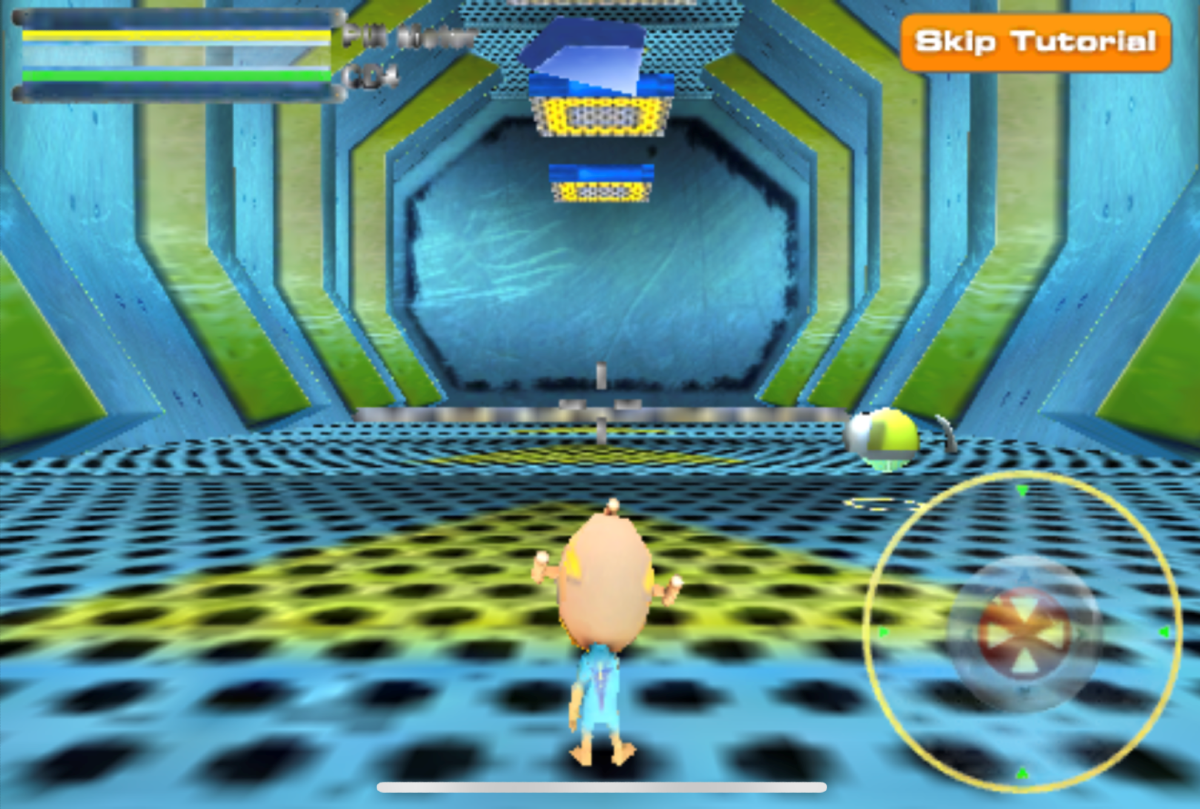
BattleViro gameplay [35].

*BattleViro* [35] is a twin-stick shooter mobile game aimed at improving antiretroviral treatment adherence among young MSM in the United States. During the game, players control an avatar that is shrunken down to fight viruses and other infections in 6 levels of increasing challenge. Each level is set on a specific organ ranging from the lungs to the brain. Throughout the different levels, the player shoots down threats to the host’s body while picking up health points in the form of medicine (Figure 3). The character also receives messages from health care personnel, encouraging them to carry on and providing clues in challenging areas of the run. In addition, the player might answer quizzes from clinician avatars to earn additional points or powers. Wrong answers are corrected and explained. In addition to the game, participants with perfect adherence would receive congratulatory texts, whereas the other participants would receive motivational messages encouraging them to carry on.

#### Viral Combat

*Viral Combat* [37] was developed by the same team that created *BattleViro*. However, instead of targeting people already with HIV, this game attempts to promote PrEP adherence. The levels are slightly different, as are the messages received by physicians and nurses; however, the main mechanics are similar. The game includes quizzes that go beyond PrEP adherence, including information on HIV and other STIs.

#### MyPEEPS

*MyPEEPS* [38-40] is a role-playing game in which young MSM with little to no sexual experience go through different scenarios guided by 4 characters (the *peeps*) who teach them about sexual health care. The game comprises 4 sequential modules (PEEPScapades). The completion of the different modules is incentivized by in-app trophies.

#### First-person Scenario Game

**Figure 4:**
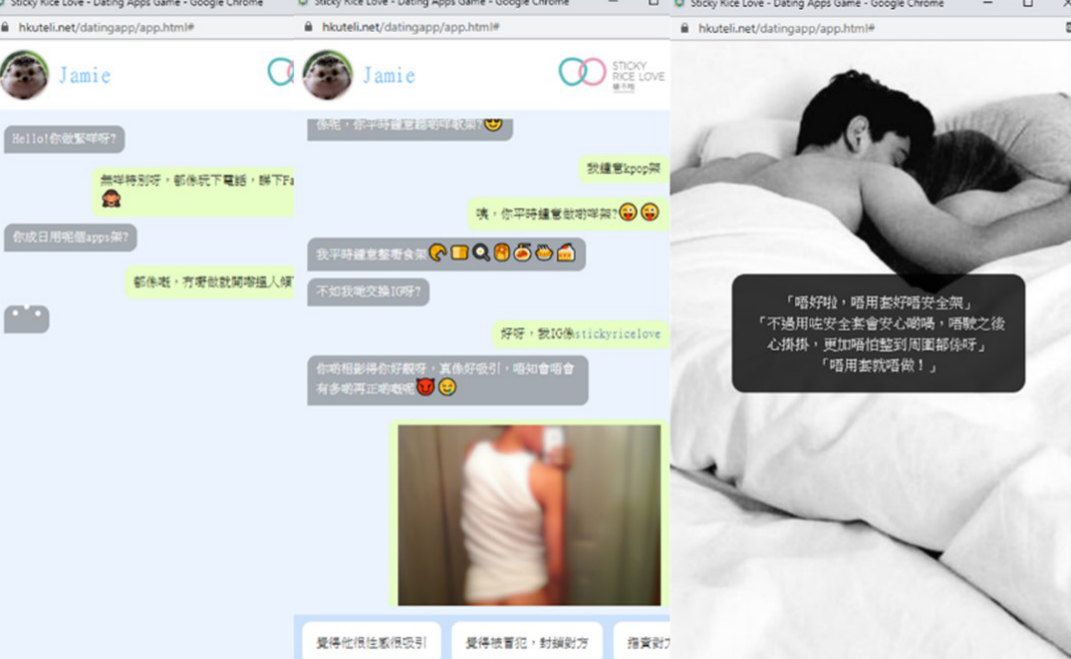
First-person scenario game example screens [41] (translation available in Multimedia Appendix 2).

First-person scenario game (*FPSG*) is the name we have provided for a multi-method intervention that aims to protect university students from the risks of using dating apps. The intervention comprises short informative videos in which students are taught about different risks, such as sexual abuse and scams. It includes a first-person simulation game in which the participant is presented with multiple choices when faced with real-life scenarios (Figure 4). The game was designed with various algorithms that resulted in positive or adverse outcomes, depending on the character choices.

### Results of Studies

In the following sections, we discuss the main results of our review, structured around the findings related to the target populations, interventions, outcomes, and study designs.

#### Target Population

Finding games that were targeted exclusively at youth aged between 15 and 25 years was not an easy task. Several identified interventions included some participants outside our selected age bracket, especially at the younger end of the age range. This was likely due, in part, to the physical location where the interventions were delivered. For example, several interventions were conducted in high schools [27-29], including students from both within and outside our age group. We did not find many studies that included participants outside the upper end of our age range. We found only 1 trial in which recruitment was done in colleges; however, even in this particular setting, the number of students aged >25 years (our upper age limit) was small [41].

Concerning gender and sexual orientation, many recent studies have focused on MSM and the prevention of STIs. A total of 7 studies had been conducted over the past 10 years; 5 of these focused exclusively on MSM. All but one of the games targeted urban youth; only 1 focused on rural populations [29].

On the basis of target population profiles, we divided the studies into 2 broad categories. First, there were those that were based in an educational institution (high school or university), included people of all genders and sexual orientations, and focused on contraception and STIs [27-29,41,42]. The second group was most commonly recruited from youth centers or sexual health clinics, focused exclusively on MSM, and had a clear focus on STI prevention and management [30,32,34,35,37,43,44].

#### Interventions

##### Overview

In the following sections, we describe the elements of the 11 games. However, first, it is important to note how little information about the games themselves was readily available in published articles. Authors often devoted little space to describing how the games looked and how they were played. Considering that playing games is a visual activity, the inclusion of images could have been a valuable way of providing this information. However, with some noteworthy exceptions [27,30,35,41], this was rarely performed; most articles did not include any form of visual aid to explain the game. Some articles included links to demos or webpages where the content was said to be available; however, in >1 case, the links were broken, or the page had already ceased to exist. Owing to the rapid nature of this review, no author and game developers were contacted during the process.

##### Game Age

Regarding the age of the games themselves, we identified a clear bimodal distribution; 3 games were created in the wake of the digital revolution ≥30 years ago, whereas the remaining group was developed more recently. Earlier games were graphically simpler but in no way less informative. We found it striking that from very early on, video games were seen as valuable tools to promote learning and attitude change.

##### Gaming Platform

The interventions used different platforms to host the games. Considering the time span across which the games were developed, it is not surprising that the most commonly used platform was that with the longest history, the PC. The changes in the games reflect the technological evolution that has affected this platform. Although popular, tablets were only mentioned in one of the games [33]. As might be expected, when mobile phones became widespread, the idea of using smartphones as platforms for serious gaming became an option. Of the 4 reviewed games for mobile phones, 2 (50%) were exclusively developed for the iPhone [35,37], whereas the other 2 (50%) did not specify which operating system they worked with [38,42]. These later games used mobile connectivity to increase participant engagement through push notifications and messages [35,36]. However, it is noteworthy that most interventions did not appear to use patches and updates to freshen their content, even when they were constantly connected to the internet.

##### Accessibility and Difficulty

A decision that is implicit in platform selection is accessibility. Most interventions aspire to be easily scalable. To achieve this, they must operate on an already popular and ubiquitous platform and use relatively little graphic processing power. We do not have access to the system requirements for any of the listed games. However, based on their description, it appears most operated on or below the considered average computing power at the time of their release.

With regard to game difficulty, all interventions can be placed on a continuum. On one end, there are games that provide an easy experience; that is, the game is seen as a vehicle through which information can be provided in an entertaining fashion. On the other end, we have games that provide a very demanding experience. The best example of this in the games we identified is *VODO*. The developers included a section of the game in which the player needed to answer 2 questions about sexual health. If either of these answers was incorrect, the game was over, and the player had to start all over again. This type of failed outcome was a very common scenario of text adventures at the time and motivated the player to replay the game several times, learning all its intricacies. An even more challenging aspect of these questions is that the answers were not provided in-game. The player was meant to search elsewhere for information; or even better, the topic should become a conversation starter for youth to discuss with their friends, parents, or teachers. This is an example of how a severely challenging task was used to *frustrate* the players into action.

As could be expected, these extremes in game difficulty were not without their issues. A nonchallenging game can be boring and can inhibit engagement. At the same time, a too-challenging game can tire a proportion of the players out of the intervention. In an effort to resolve these 2 positions, some game developers opted for an increasing level of difficulty; that is, instead of having a flat, low-level difficulty throughout the experience or a series of extreme spikes every so often, these games ramp up the difficulty with every level [35,37].

##### Expected Playthroughs and Playing Time

There are 2 related elements to consider here: expected playthroughs and expected contact time. In other words, how many times are players expected to play the game, and for how long? As we will discuss in the following sections, repetition, rehearsal, and feedback play an essential role in learning through games; therefore, it is expected that most games were designed to keep the player engaged in >1 run. Unfortunately, there is little information about these aspects in most of the included articles. Among the games that specified an expected playing time, we found periods of <1 hour of gaming. However, it was not always clear whether these times were for single or multiple playthroughs.

##### Type of Game and Game Setting

Game settings can be broadly divided into 2 types: realistic and science fiction. There was a clear preference for the latter in our sample of games. Most games were situated in locations and environments to which the player could directly relate, such as hanging out with friends after school or going to a house party. The idea behind this is that a greater similarity between the simulated situation and a plausible real-life event might make it more likely that players will relate to the content and act upon what they have learned. Game developers have gone into great efforts to create content grounded in reality, where situations that the players have directly experienced are portrayed both didactically and accurately. For example, *SOLVE* allowed players to personalize their avatars, and *The Baby Game* used actual prices when they calculated the costs of raising a child.

The types of games in the realistic group were highly diverse. Table 1 presents a list of the different styles of games that were included. We want to highlight 3 features. First, the variety is notable; as can be seen, games ranged from management simulators to role-playing games. The second feature is the relative preponderance of the dating simulators. Approximately 44% (4/9) of the nonfiction games were dating simulators. However, even within this specific setting, we found different styles (eg, text adventures, 3D, and chat simulators). Finally, the role of complementary activities in each game should be mentioned. Minigames are found in several games, particularly in the form of quizzes [29,34,36,37]. This seems to have been one of the main strategies through which game developers delivered specific sexual health knowledge.

However, not all games followed a realistic route. *BattleViro* and *Viral Combat* opted instead for science fiction in the action setting [35,37]. In both games, the characters are shrunk down to a microscopic scale and are meant to protect the human body from infections by shooting down viruses, bacteria, and vectors of disease. Here, the developers tried to create a power fantasy in which the player can take control of their actions and reach a desirable healthy state. The content and settings were still related to the topic of focus (antiretroviral treatment and PrEP adherence); however, the developers avoided making direct or explicit statements about them.

We can see that there is >1 valid strategy for promoting engagement with the material. One school of thought aims to create easy-to-relate experiences, whereas the other uses a fast-paced game to empower participants into action.

##### Single or Multiplayer Game (Private or Social Experiences)

An element mentioned throughout the different interventions is the contrast between creating a single or multiplayer experience. In other words, was the game designed to be played alone or with a group? Most of the games reviewed appear to have been designed for single-player use. However, it should be noted that all single-player experiences can be turned into multiplayer experiences by the players themselves. Researchers reported that, in several cases, games that were not meant to be social experiences were transformed into a group activity when a player spontaneously brought their friends or partner to play the game together and comment on it.

The decision to develop a single- or multiplayer game is affected by several factors. The first is the target population. Interventions focused on MSM were very keen on not *outing* their players involuntarily or having them openly disclose their health information; hence, single-player games might have been preferred. Second, the game’s topic of focus is an important factor to consider. Some topics are easier to work with at the individual level than at the group level. For example, *SOLVE* was a game that tried to decrease the feelings of shame that gay or bisexual men might experience regarding their sexual preferences [35]. Considering that many players had strong feelings of shame and were reticent to disclose information about their sexual interests, they may not have been comfortable playing a game with others.

The third influence is logistical, technological, or economic restriction. When PCs were not ordinary household items but specialized pieces of hardware, they were not as commonly available as they are now. For this reason, older games tended to be a social experience; many people had to use the same computer to make it viable for enough players to play the game [27,28].

The final reason for choosing single- or multiplayer games relates to the learning strategy of choice. Some game developers opted to purposely promote out-of-game discussions of sexual health topics [28]. The aim was to make the game a topic for discussion with family members, teachers, and friends.

##### Outcome Change Mechanisms

There are several ways in which we could try to classify the underlying mechanisms used in video games to change specific behaviors in users. Here, we divided the mechanisms into 3 categories: those based on knowledge, those focused on enhancing skills and self-efficacy, and those that motivate change through emotions. These groups are not mutually exclusive; 1 intervention might have >1 underlying mechanism.

Some games aim to provide knowledge, expecting that it will generate behavior change. For example, some games share facts about contraceptives, their efficacy, and the risks involved in not using them. In such cases, one of the most critical elements is to provide a clear and easy-to-understand message. It has been noted that most interventions try to make the message grounded in a specific element or situation in the game. Many games focusing on providing knowledge prioritized the provision of immediate and clear feedback, specifying where and when an error was made and what its potential outcomes might be. The same applies to decisions that have a positive outcome. For example, in *SOLVE*, when a player chose to engage (or not) in in-game sex, they were offered a quick recap of all the previous decisions that drove them to their current state (decisions that were not always apparent at the time they were made).

The final element of knowledge is how it is constructed. We have previously stated that the message must be clearly stated. However, for some interventions, this did not necessarily mean that the message had to be explicitly delivered. For example, the *HIV risk game* had a clear message that needed to be delivered: older people were more likely to have HIV than younger people. Players played 10 rounds of the game in which they made a judgment about which character was more likely to have HIV. As feedback, the players did not receive the correct answer; they only knew whether they were right or wrong. This key message was supposed to be inferred (constructed) by the participants based on their in-game experience.

Two of the most frequently used strategies in games focused on increasing the participants’ skills and self-efficacy, which was achieved by a mixture of relatability and rehearsal. By relatability, we mean all the different factors that can make the situation in a game similar to the ones players face or think they will face. The developers made great efforts to provide experiences grounded in those that the players have had or will experience. The assumption is that, in general, the closer a setting and its characters are to the real world, the easier it will be for the player to assimilate the lesson and put it into practice. This is one of the reasons why several games designed characters with different personalities and stories so that the player can easily associate 1 or several of them with their friends and acquaintances. Similarly, one of the reasons why some avatars were customizable was to make it easier for players to empathize with their in-game presence. The same can be said of the setting in which the interactions occur. In several cases, the setting was very similar to that currently experienced by the players. One of the clearest cases of relatability is in the FPSG game. The player learns about the risks of dating apps by playing a game that uses an instant messaging app as one of its primary interfaces.

Similarly, rehearsal and repetition also played a significant role in improving self-efficacy. The idea is that players will train themselves to make safe decisions in real life because they have made the same correct decisions in a virtual world before. The more times a player does something, the more likely it is for him or her to feel (and be) proficient in it.

The final strategy relies on using emotions to generate a reaction in the player. There are several methods in which this has been performed in different games. Some developers opted to generate negative emotions that frustrated or scared players into action. For example, in *Romance*, if the players initiated unprotected sex, they would have a baby that would cry randomly during the game, negatively affecting their relationships with their friends. Other games used positive emotions to inspire players to act. *BattleViro* and *Viral Combat* are good examples of this practice, having used fast action, increasingly challenging shooter-style games to empower their players to take control of their treatment. Finally, there were games that aimed to reduce the negative emotions that inhibit players’ ability to do something. The best example of this practice is *SOLVE*, a game whose main aim was to reduce the feelings of shame that MSM might experience. Through a series of stories and vignettes, the intention was that the player might consciously acknowledge their desires as something normal, which carries no stigma.

##### Game Development

We cover 3 main topics in this section. We begin with a general description of the development process of the games. We then assess the involvement of stakeholders in the creation of the game: who was invited, when, and in what capacity.

###### Development Process

The published papers provided little information on the development of game mechanics. There was often no data regarding how long the game design lasted, how much its budget was, who and how many people were involved, and what program or programs and engine or engines they used to create it. From conversations with researchers, we know that in some cases, university-based groups were in charge of software development. However, apart from *FPSG*, very little additional information is readily available from these articles or other related publications on game development.

Some interventions adapted previous activities or interventions for the construction of new games. In some cases, existing materials and activities from previous interventions were adapted to a video game form. The details of these interventions were usually left nebulous; however, we know that in the case of *MyPEEPS*, *Keep it up!*, and *SOLVE*, a considerable part of the content of the games was taken from previous non–video game–based interventions. For example, *MyPEEPS* included 4 characters (the titular *peeps*), who were a composite of previously existing characters used during the formative phase of the intervention.

The development of other interventions was probably informed by existing games, although few articles provided much detail about this. The only exception to this trend was *Viral Combat*, heavily influenced by *BattleViro* [37]. The same team of researchers developed both games, and one might even say that the former is an improved version of the latter.

In summary, we found that interventions have either been developed entirely from scratch or based on a previous in-person intervention. Explicit references to previously existing games were unusual in the reviewed studies.

###### Stakeholder Participation

The teams in charge of designing the interventions frequently made considerable efforts to involve different stakeholders throughout the process. Among the stakeholders who participated in the design of games were end users [32,35,41], members of nongovernmental organizations concerned with sexual health or youth well-being [34], and unspecified community leaders [28]. There is little to no mention of the involvement of parents, teachers, or other authority figures. Focus groups [34] and in-depth interviews [32,35] were used to access stakeholders’ views.

There were 3 main reasons for stakeholder involvement. The first reason was to conduct a needs assessment. This allowed the intervention designers to prioritize topics or behaviors that required specific attention. For example, in the development of the *FPSG* intervention, 4 focus groups were held by the developer to identify key risks that caused concern among young people using dating apps in Hong Kong. The second reason to involve stakeholders was to improve the quality of the game itself. For example, *Keep it up!* conducted interviews with stakeholders to ensure that the situations and languages they used in their club games were similar to those experienced by young men in their everyday interactions [33]. This allowed them to generate greater engagement with the final users by presenting situations comparable with those they had experienced previously. Finally, approval from the governing body is needed. By involving community leaders and local authorities, intervention designers could ensure that they would receive support for the subsequent stages of the process. For example, *VODO* involved people from 30 different local institutions to avoid the inclusion of content or situations that might have been perceived as unacceptable by the community [28].

##### Multi-Method Interventions

A final element to discuss is that although all interventions relied considerably on video games to achieve their goals, it was not necessarily the only method they used. Approximately 27% (3/11) of the games were meant to be played in conjunction with other activities.

For these specific interventions, the games seem to be one of the few activities in which the participants could take agency and act upon the knowledge they received. For example, when the participants are completing scales or watching videos, they are fairly passive, and the moments in which they play the games are the only times when they really take control, make decisions, and see their results. Although no intervention specified the playing time, or the time used in the other modules, it appeared that the games were the activities that comprised most of the participants’ time.

#### Outcomes

##### Overview

In this section, we discuss the effectiveness of the interventions in achieving their goals, organized by the outcomes adopted in our search criteria. Multimedia Appendix 3 provides a summary of the results.

##### Decrease in Unintended Pregnancies

Rather unexpectedly, none of the studies assessed the number of pregnancies. There are 2 possible explanations for this. First, almost half of the chosen games were not marketed to women but to MSM. Second, the sample sizes were too small, and the follow-up periods were too short, for the relatively low occurrence of pregnancy to become a viable measure of the success of an intervention.

##### Decrease in STIs

Only *Keep it up!* used STI biomarkers to assess changes in STI incidence. The researchers tested for chlamydia and gonorrhea through self-collection of rectal swabs. Through matched odds ratios, the control group showed a 55% increase in STI incidence, whereas the treatment group showed a decrease of 51%. These results were significant; however, we must be mindful that this intervention had multiple components, and the video game was only one of them.

##### Increased Contraceptive Use

Approximately 27% (3/11) of interventions measured changes in reported contraceptive use. Unfortunately, they did so in very different ways, which limited our ability to compare them. *Choose Your Own Adventure* asked about condom use at the last intercourse and found no effect of the intervention. The other 2 studies assessed the frequency of UAI with nonprimary partners during the past 3 months. Although *SOLVE* was unable to show significant differences between the treatment and control groups, *Keep it up!* reported a significant decrease in the number of UAI events 1 year after the start of the intervention.

In summary, studies on the effects of video game interventions on contraceptive use have shown inconsistent results.

##### Acquisition of New Knowledge Regarding Sexual Health

The acquisition of new knowledge was one of the most commonly measured outcomes; however, the topics and measures varied significantly among the different interventions. Frequently, ad hoc questionnaires were created to assess differences between the treatment and control groups. *The Baby Game* quizzed participants on the costs (both time and money) involved in taking care of a baby. *Romance* used the same methodology but compared knowledge about the efficacy of different contraceptive methods. Studies on both of these interventions suggested improved knowledge in the treatment group compared with the control group. However, we should keep in mind that knowledge was assessed only immediately after the game ended and that no effect size measure was presented. Other interventions also relied on ad hoc tests; however, it was unclear exactly what topics they explored. *Choose Your Own Adventure* showed positive results (of medium effect size); however, *VODO* failed to do so. Both interventions followed a pretest-posttest design.

A comparable example is that of *BattleViro* and *Viral Combat.* They both tested their participants’ HIV knowledge, and although they were very similar games, only *BattleViro* showed positive results. It should be noted that *BattleViro* tested their participants 16 weeks after the intervention started, whereas *Viral Combat* did so at weeks 12 and 24. The extended period between intervention and data collection in *Viral Combat*, compounded by attrition of 32% of the original sample, might have biased the intervention results. However, one would also expect that participants who stayed longer would be more engaged, would have clocked in more hours in the game, and would have a better overall performance.

In conclusion, we do not have enough evidence to clearly state that games have a significant effect on increasing knowledge of sexual health topics. The variety of topics assessed, the limited amount of information regarding the content of the tests, and the large variability in the time between intervention and postintervention assessment precludes our ability to establish a clear causal relationship between playing and learning.

##### Changes in Perceived Risk of Pregnancy

Only 2 games assessed this variable. *Romance* asked participants to assess the odds of becoming pregnant when having unprotected sex. Improvements in favor of the treatment group immediately after they finished playing the game were reported. Unexpectedly, a study on *Choose Your Own Adventure* found significant differences in favor of the control group. However, the researchers measured a construct called *susceptibility*, which merged the perceived risk of pregnancy with the perceived risk of STIs; thus, there might have been some cross-contamination in the assessment. The authors speculated that their results might be related to the fact that fewer people initiated sexual activities in the treatment group (ie, they were abstinent) than in the control group; hence, they did not feel at risk of any adverse outcomes related to having sex. They also considered that as the treatment group was more aware of the risks and the measures they could take against them, they felt better able to protect themselves.

##### Changes in Perceived Risk of STIs

Approximately 36% (4/11) of games addressed this topic. We have already discussed the findings of *Choose Your Own Adventure*. The entire intervention of the *HIV risk game* was centered on assessing the risk of someone having HIV based on their age and gender. Positive results were obtained for both men and women. The assessment was performed immediately after the intervention and 3 months later (the last time only for male participants).

*BattleViro* and *MyPEEPS* also assessed the perceived risk of STIs but in an indirect fashion by asking about STI testing. *BattleViro* measured the types of sexual behavior, frequency of sex, and number and gender of partners reported in the past 3 months. The authors found no differences between the control and treatment groups. *MyPEEPS* measured the frequency of STI testing and found that after the intervention, those in the treatment group were more likely to get tested than those in the control group.

##### Attitudinal Change Toward Safe Sex

Approximately 18% (2/11) of interventions targeted attitudinal changes toward safe sex. *VODO* measured participants’ attitudes toward sex on 2 axes: liberal versus conservative and positive versus negative. Both the control and treatment groups shifted to a more liberal position; however, the change was greater in the treatment group. The change in this group was sufficient for it to move, on average, from a conservative perspective toward a liberal one. No significant changes were observed in the positive and negative axes.

*Choose Your Own Adventure* assessed the predisposition toward waiting to have sex and found a significant effect of their intervention among students aged between 15 and 16 years. After completing ≥1 of its modules, participants were more likely than those in the control group to postpone sexual initiation.

##### Self-efficacy Toward Sexual Health

Self-efficacy, be it general or specific to sexuality, was one of the most frequently chosen outcome variables across the different games. *Choose Your Own Adventure* considered 4 domains of sexual self-efficacy: condom negotiation self-efficacy, condom use self-efficacy, situational self-efficacy (the ability to control a situation that might be conducive to sex), and refusal self-efficacy (the ability to say no to sexual intercourse). However, the findings were mixed. The intervention increased participants’ self-efficacy toward condom negotiation and situational self-efficacy; however, no effects were found regarding condom use or refusal. It is not surprising that condom use self-efficacy did not change considerably between groups as the intervention did not include any components that directly taught students how to apply and use a condom. However, it did have 1 activity specifically focused on improving refusal skills, which was tightly tied to the game itself. The fact that this activity failed to produce the desired results for this variable is noteworthy.

*BattleViro* and *Viral Combat* assessed a similar domain of self-efficacy: participants’ belief that they would be able to adhere to a treatment regime. Both interventions found no significant change in either the short (12 weeks) or long-term (16 weeks and 24 weeks) assessments. *MyPEEPS* reported positive results when assessing HIV self-efficacy in the short term (3 months). Researchers have yet to publish their results for the long-term assessments (6 months).

We consider that the results on self-efficacy are mixed. Considering that the interventions are varied in methodology and topics and that they have worked on different domains of self-efficacy, this is not a particularly surprising result.

##### Decrease in the Number of Sexual Partners

Approximately 27% (3/11) of interventions aimed at reducing the number of sexual partners, all of them for MSM—*Keep it up!, Viral Combat,* and *MyPEEPS*—and none resulted in a reduction in the number of sexual partners in their samples.

##### Increase in Adherence to Prophylaxis or Treatment

Approximately 27% (3/11) of interventions focused on increasing adherence to either treatment or PrEP. *BattleViro* and *Viral Combat* used a mixture of bioindicators, self-reported behavior, and electronic device follow-ups to assess this outcome. A total of 2 bioindicators were used: HIV-1 viral load in *BattleViro* and 1ARV (activator protein 1) levels in *Viral Combat*. *BattleViro* produced equivalent decreases in HIV-1 viral load in both treatment and control groups. *Viral Combat* reported results that favored the treatment group at both 12 weeks and 24 weeks after the beginning of the intervention; however, these findings were not statistically significant. Treatment adherence, measured by self-report in *Viral Combat* and by self-report plus electronic device records in *BattleViro*, showed similar results. In this regard, no intervention showed better results than the usual treatment. *MyPEEPS* also measured PrEP and postexposure prophylaxis adherence using self-reported measures and found no significant differences between the treatment and control groups.

#### Study Designs

Bearing in mind that our search criteria only allowed for RCTs and quasi-RCTs, we identified 2 main study designs. Studies were either posttest-only trials [31,37,43] or pretest-posttest trials [27-30,33,35,36,42].

Depending on the study, the control group received different treatments. Waiting-list control was one of the most straightforward control designs. A more complex one was treatment as usual (TAU), where the usual or standard was given to a group of participants.

For example, for *The Baby Game*, researchers compared their game with a regular sexual health education class for that specific age group [28]. A similar option was TAU+. Here, the participants received TAU and an additional component that was functionally similar but thematically different from the experimental group. For example, as *BattleViro* provided smartphones so that participants could play the game, they also provided smartphones to the control group. However, these iPhones did not have the specific game installed but another non–HIV-related game [35]. Another form of control group provided more or less the same content as the game but in a delivery mode that had no ludic or interactive elements. For example, *Keep it up!* provided an internet-based experience with the same information as their intervention but using static slides instead of the more dynamic approach taken with the treatment group.

Finally, in reviewing whether the studies had adequate sample sizes, we found that the sample sizes were generally large enough to detect expected differences. Most studies, especially the more recent ones, determined their sample size based on a power analysis (although this analysis was usually constructed around educated guesses). Even if the sample size in the reviewed studies was usually large enough, one of the main threats to statistical power was a relatively large attrition rate, especially among studies with multiple or long follow-ups. The most extreme case was in the *HIV risk game* study, which reported an attrition rate of 66.8%. In the remaining studies, the attrition rate was approximately 30%.

## Discussion

### Principal Findings

The findings of our review yielded important conclusions and implications for future research and game development. First, the findings highlighted considerable diversity in video game–based interventions. Although all of them addressed similar topics, they did so in fairly distinct ways. The outcomes assessed in studies evaluating games were also very diverse and, even when similar, were measured differently in each study. Second, we found that game developers have made great efforts to elicit experiences tailored to the specific needs of the targeted population, most often achieving this through regular stakeholder participation activities throughout the game development process.

One of the most surprising findings was the age of the identified games. The fact that the games were developed over such an extended period suggests that even when the graphical complexity and the interface changed considerably, learning through gaming is and has been seen as a viable and successful strategy.

However, although game interventions for sexual health have been in existence for almost three decades, relatively few studies have evaluated them, and the results of previous studies have been mixed. Moreover, there is little clarity regarding which specific elements of a game facilitate a positive outcome. This is partly because of the diversity of the behavior change mechanisms underlying interventions, the variety of the games themselves, the populations they target, the outcomes measured, and how these are measured. All these differences make it challenging to identify a clear causal link between playing a game and improving an aspect of sexual health. However, although the impact on sexual health is not always clear, the fact that video game–based interventions are of interest to most young people is well-established.

Nevertheless, there are other less positive aspects of research in this area that we need to acknowledge. First, there is a lack of information available in published reports on different games, especially in the gameplay aspect. This ties to another unexpected finding of our review. Although video game–based interventions are meant to be easily scalable, there are no reports on any of these games being picked up for broad distribution. In fact, only *BattleViro* was readily available for download. Moreover, although the interventions were very varied, almost all of them targeted people living in urban areas of the United States. There were no interventions developed with populations from lower-income countries in mind, and almost no interventions were aimed at rural populations. Finally, very few interventions were informed by a behavioral theory or model.

### Strengths and Limitations

Our review had some notable strengths. We followed the Cochrane Rapid Reviews Method Group guidelines [22,24] to conduct the review and searched several key literature databases. A second reviewer was involved in screening 20% (51/257) of the articles at the title and abstract screening stage and 50% (35/70) of the articles at the full-text screening stage.

Some limitations of our review should also be acknowledged. As this review was rapid, our search used 5 databases; thus, we may not have identified all the relevant literature. We restricted our age range to 15 to 25 years and, in the screening process, noticed that some interesting game-based interventions focused on younger adolescents and children. A final limitation was that we did not exclude studies based on quality.

### Conclusions and Recommendations

In conclusion, we do not have enough evidence to clearly state that games have a significant effect on sexual health among young people. The interventions and how they were evaluated were too diverse to reach a clear conclusion. However, based on the original authors’ criteria for success, we have compiled a set of recommendations for developing game-based interventions to improve sexual health in young people (Textbox 1).

**Recommendations for developing game-based interventions to improve sexual health in young people.**

**Recommendations for developing game-based interventions to improve sexual health**

- Stakeholders should be involved in different stages of the game development process. Most successful strategies used qualitative participatory methods involving multiple stakeholders.
- A pilot phase in the development of games is strongly encouraged. This enables specific elements that could otherwise jeopardize the success of the initiative to be identified and modified.
- One of the most crucial decisions during the game development process is whether intervention participants are expected to go through the game once or multiple times. This affects the length, difficulty setting, and the main mechanics of the game.
- There are several viable ways in which a game can try to change someone’s behavior. None have proven to be markedly better than the others. However, some recommendations are as follows:
  Knowledge-based interventions should aim to provide a clear message, and this message does not need to be explicit. In fact, some researchers recommend that the message is not explicitly stated but constructed by the players themselves.
  Self-efficacy and skill-building interventions aim to provide easily relatable experiences and those that feel proximal to the player. The closer the player feels a game experience is to their own experience, the more likely it is that they will act upon it. The game serves as a rehearsal for the decisions they will make in real life.
- Disregarding the mechanisms chosen by developers, some common elements are shared by most strategies:
  Feedback is better if it is clear, detailed, and immediate. When playing, it is encouraged that users recap their decisions and learn which actions drove them to their current stage (whether positive or negative).
  Repetition (as long as it does not transform into tediousness) is usually favorable, especially for skill building and knowledge acquisition.
- There are 3 common threats that plague these interventions:
  Lack of technical support, especially after the game development phase ends, is a common threat.
  Another threat is the stagnation of the content; that is, no updates are provided, and no new content is delivered.
  The games are not easily found when someone wants to use them in other contexts. The created game should be openly available on the web if possible.
- Game quality indicators (including playing time) were registered using self-reported measures. A suitable workaround using in-game data collection is recommended to bypass social desirability and recall issues that affect purely self-reported information.
- A plan for the implementation of the intervention should be made at the early stage of the project.

## Appendix

Multimedia Appendix 1 Search strategy.

Multimedia Appendix 2 First-person scenario game example—English translation.

Multimedia Appendix 3 Summary of results per intervention.

## Abbreviations

FPSG: first-person scenario game
MSM: men who have sex with men
NPC: nonplayer character
PrEP: pre-exposure prophylaxis
RCT: randomized controlled trial
SOLVE: Socially Optimized Learning in Virtual Environments
STI: sexually transmitted infection
TAU: treatment as usual
UAI: unprotected anal intercourse
