# Supplementary material for: Using Video Games to Improve the Sexual Health of Young People Aged 15 to 25 Years: Rapid Review
Source: JMIR Serious Games. 2022 May 19;10(2):e33207. doi: 10.2196/33207 (PMC9164099; doi:10.2196/33207)
Supplement: Multimedia Appendix 1 [file games_v10i2e33207_app1.pdf]

I. Search strategies

Date Run: 18/03/2021 21:49:25

CENTRAL search strategy

ID Search Hits
#1 MeSH descriptor: [Young Adult] explode all trees 66109
#2 MeSH descriptor: [Adolescent] explode all trees 104818
#3 (adolescent* OR teenager* OR teen* OR youth* OR young adult* OR young m?n OR young wom?n):ti,ab,kw 198567
#4 #1 OR #2 OR #3 198567
#5 MeSH descriptor: [Sexual Health] explode all trees 49
#6 MeSH descriptor: [Reproductive Health] explode all trees 82
#7 MeSH descriptor: [Contraception] explode all trees 475
#8 MeSH descriptor: [Pregnancy, Unwanted] explode all trees 44
#9 MeSH descriptor: [Pregnancy, Unplanned] explode all trees 86
#10 MeSH descriptor: [Sexually Transmitted Diseases] explode all trees 12224
#11 (Contracept* OR "family planning" OR pregnan* OR Sexually transmitted disease* OR Sexually transmitted infection* OR STI OR STD):ti,ab,kw 78109
#12 {OR #5-#11} 87974
#13 MeSH descriptor: [Video Games] explode all trees 706
#14 MeSH descriptor: [Mobile Applications] explode all trees 722
#15 MeSH descriptor: [Games, Experimental] explode all trees 125
#16 (videogame* OR video game* OR electronic game* OR video-game* OR computer game* OR game* OR gaming):ti,kw,ab 6640
#17 {OR #13-#16} 7324
#18 #4 AND #12 AND #17 in Trials 94

Embase search strategy

(‘young adult'/exp OR ‘adult, young’:ti,ab,kw OR ‘prime adult’:ti,ab,kw OR ‘prime adults’:ti,ab,kw OR ‘young adult’:ti,ab,kw OR ‘young adults’:ti,ab,kw OR ‘adolescent'/exp OR ‘adolescent’:ti,ab,kw OR ‘teenager’:ti,ab,kw OR ‘juvenile'/exp OR ‘juvenile’:ti,ab,kw OR ‘youth’:ti,ab,kw OR ‘student'/exp OR ‘apprentice’:ti,ab,kw OR ‘student’:ti,ab,kw OR ‘students’:ti,ab,kw OR ‘trainee’:ti,ab,kw OR adolescent*:ti,ab,kw OR teen*:ti,ab,kw OR youth*:ti,ab,kw OR ‘young m#n’:ti,ab,kw OR ‘young wom#n’:ti,ab,kw OR student*:ti,ab,kw)
AND
(‘video game'/exp OR ‘tv games’:ti,ab,kw OR ‘computer game’:ti,ab,kw OR ‘computergame’:ti,ab,kw OR ‘television game’:ti,ab,kw OR ‘video game’:ti,ab,kw OR ‘video games’:ti,ab,kw OR ‘videogame’:ti,ab,kw OR ‘videogames’:ti,ab,kw OR ‘mobile application'/exp OR ‘mobile app’:ti,ab,kw OR ‘mobile application’:ti,ab,kw OR ‘mobile applications’:ti,ab,kw OR ‘mobile apps’:ti,ab,kw OR ‘portable software app’:ti,ab,kw OR ‘portable software application’:ti,ab,kw OR ‘portable software applications’:ti,ab,kw OR ‘portable software apps’:ti,ab,kw OR ‘tablet application’:ti,ab,kw OR ‘game'/exp OR ‘game’:ti,ab,kw OR ‘game model’:ti,ab,kw OR ‘game theory’:ti,ab,kw OR ‘games, experimental’:ti,ab,kw OR ‘model, game’:ti,ab,kw OR videogame* OR ‘video game*’:ti,ab,kw OR ‘computer game*’:ti,ab,kw OR ‘mobile game*’:ti,ab,kw OR ‘mobile app*’:ti,ab,kw OR ‘cellphone app*:ti,ab,kw’ OR game:ti,ab,kw)
AND
(‘sexual health'/exp OR ‘reproductive health'/exp OR ‘family planning'/exp OR ‘birth interval’:ti,ab,kw OR ‘birth intervals’:ti,ab,kw OR ‘family building’:ti,ab,kw OR ‘family planning’:ti,ab,kw OR ‘family planning clinic’:ti,ab,kw OR ‘family planning service’:ti,ab,kw OR ‘family planning services’:ti,ab,kw OR ‘interval, birth’:ti,ab,kw OR ‘natural family planning’:ti,ab,kw OR ‘natural family planning methods’:ti,ab,kw OR ‘safe sex'/exp OR ‘safe sex’:ti,ab,kw OR ‘safer sex’:ti,ab,kw OR ‘unplanned pregnancy'/exp OR ‘mistimed pregnancy’:ti,ab,kw OR ‘pregnancy, unplanned’:ti,ab,kw OR ‘unintended pregnancies’:ti,ab,kw OR ‘unintended pregnancy’:ti,ab,kw OR ‘unplanned pregnancies’:ti,ab,kw OR ‘unplanned pregnancy’:ti,ab,kw OR ‘unwanted pregnancy'/exp OR ‘pregnancy, unwanted’:ti,ab,kw OR ‘undesired pregnancies’:ti,ab,kw OR ‘undesired pregnancy’:ti,ab,kw OR ‘unwanted pregnancies’:ti,ab,kw OR ‘unwanted pregnancy’:ti,ab,kw OR ‘sexually transmitted disease'/exp OR ‘bacterial sexually transmitted diseases’:ti,ab,kw OR ‘sexually transmitted disease’:ti,ab,kw OR ‘sexually transmitted diseases’:ti,ab,kw OR ‘sexually transmitted diseases, bacterial’:ti,ab,kw OR ‘sexually transmitted diseases, viral’:ti,ab,kw OR ‘sexually transmitted infection’:ti,ab,kw OR ‘std’:ti,ab,kw OR ‘vd’:ti,ab,kw OR ‘veneral disease’:ti,ab,kw OR ‘venereal disease’:ti,ab,kw OR ‘venereal infection’:ti,ab,kw OR ‘viral sexually transmitted diseases’:ti,ab,kw OR ‘contraception'/exp OR ‘abstinence, periodical’:ti,ab,kw OR ‘anticonception’:ti,ab,kw OR ‘antifertility’:ti,ab,kw OR ‘conception control’:ti,ab,kw OR ‘contraception’:ti,ab,kw OR ‘contraceptive technique’:ti,ab,kw OR ‘female contraception’:ti,ab,kw OR ‘male contraception’:ti,ab,kw OR ‘periodic abstinence’:ti,ab,kw OR ‘periodic continence’:ti,ab,kw OR ‘periodical abstinence’:ti,ab,kw OR ‘rhythm method’:ti,ab,kw OR ‘sterilisation, involuntary’:ti,ab,kw OR ‘sterilisation, reproductive’:ti,ab,kw OR ‘sterilisation, sexual’:ti,ab,kw OR ‘sexual health’:ti,ab,kw OR ‘reproductive health’:ti,ab,kw OR ‘safe adj sex’:ti,ab,kw OR ‘unsafe adj sex’:ti,ab,kw OR ‘unwanted pregnan*’:ti,ab,kw OR ‘unplanned pregnan*’:ti,ab,kw OR ‘unintended pregnan*’:ti,ab,kw OR condom*:ti,ab,kw OR contracept*:ti,ab,kw)
AND
(‘randomized controlled trial'/exp OR ‘controlled trial, randomized’ OR ‘randomised controlled study’ OR ‘randomised controlled trial’ OR ‘randomized controlled study’ OR ‘randomized controlled trial’ OR ‘trial, randomized controlled’ OR ‘controlled clinical trial (topic)’/exp OR ‘controlled clinical trial (topic)’ OR ‘controlled clinical trials’ OR ‘controlled clinical trials as topic’ OR ‘non randomized controlled trials as

topic’ OR ‘non-randomized controlled trials as topic’ OR ‘experimental design'/exp OR ‘doe approach’ OR ‘design of experiment’ OR ‘experimental design')

Medline search strategy (Via PubMed)

((adult, young[MeSH Terms]) OR (adolescent[MeSH Terms]) OR (adolescent*[Title/Abstract]) OR (teen*[Title/Abstract]) OR (youth*[Title/Abstract]) OR (young men[Title/Abstract]) OR (young women[Title/Abstract]))

AND

((sexual health[MeSH Terms]) OR (reproductive rights[MeSH Terms]) OR (family planning[MeSH Terms]) OR (contraception[MeSH Terms]) OR (adolescent pregnancy[MeSH Terms]) OR (pregnancies, unplanned[MeSH Terms]) OR (pregnancies, unwanted[MeSH Terms]) OR (disease, sexually transmitted[MeSH Terms]) OR (reproductive health[MeSH Terms]) OR (sexually transmitted disease*[Title/Abstract]) OR (sexually transmitted infection*[Title/Abstract]) OR (sexual health[Title/Abstract]) OR (reproductive rights[Title/Abstract]) OR (contracept*[Title/Abstract]) OR (condom*[Title/Abstract]) OR (unplanned pregnanc*[Title/Abstract]) OR (unwanted pregnanc*[Title/Abstract]) OR (STI[Title/Abstract]) OR (STD[Title/Abstract]) OR (sexually transmitted disease*[Title/Abstract]) OR (sexually transmitted infection*[Title/Abstract]) OR (safe sex*[Title/Abstract]) OR (safe-sex[Title/Abstract]))

AND

((video game[MeSH Terms]) OR (experimental game[MeSH Terms]) OR (mobile applications[MeSH Terms]) OR (game*[Title/Abstract]) OR (video-game*[Title/Abstract]) OR (videogame*[Title/Abstract]) OR (video game*[Title/Abstract]) OR (electronic game*[Title/Abstract]) OR (computer game*[Title/Abstract]) OR (mobile game*[Title/Abstract]) OR (cellphone game*[Title/Abstract]))

AND

((controlled clinical trials, randomized[MeSH Terms]) OR (controlled clinical trial[MeSH Terms]) OR (experiment*[Title/Abstract]) OR (randomised controlled trial[Title/Abstract]) OR (randomized controlled trial[Title/Abstract]) OR (RCT[Title/Abstract]))

AND

(MEDLINE [subset])

PsycInfo search strategy (Via APA PsycNET)

(youth OR adolesce* OR teen* OR young people OR young adult* OR young m#n OR young wom#n OR student*)
AND
(sexual health OR reproductive health OR reproductive rights OR family planning OR contracept* OR unplanned pregnanc* OR unwanted pregnanc* OR sexually transmitted disease* OR sexually transmitted infection* OR venereal disease* OR STI OR STD OR VD OR safe#sex)
AND
(video game* OR video-game* OR computer game* OR computer-game* OR electronic game* OR mobile game* OR mobile-game* OR mobile app* OR mobile-app OR cell phone game* OR cell phone app* OR gaming)
AND
(experiment* OR randomised controlled trial OR randomized controlled trial OR RCT)

Scopus search strategy

TITLE-ABS-KEY(youth OR adolesce* OR teen* OR (young people) OR (young adult*) OR (young m?n) OR (young wom?n) OR student*)

AND

TITLE-ABS-KEY((sexual health) OR (reproductive health) OR (reproductive rights) OR (family planning) OR contracept* OR (unplanned pregnanc*) OR (unwanted pregnanc*) OR (sexually transmitted disease*) OR (sexually transmitted infection*) OR (venereal disease*) OR STI OR STD OR VD OR safe?sex)

AND

TITLE-ABS-KEY((video game*) OR (video-game*) OR (computer game*) OR computer-game* OR (electronic game*) OR (mobile game*) OR mobile-game* OR (mobile app*) OR mobile-app OR (cell phone game*) OR (cell phone app*) OR gaming)

AND

TITLE-ABS-KEY(experiment* OR (randomised controlled trial) OR (randomized controlled trial) OR RCT)
