# Supplementary material for: Using Video Games to Improve the Sexual Health of Young People Aged 15 to 25 Years: Rapid Review
Source: JMIR Serious Games. 2022 May 19;10(2):e33207. doi: 10.2196/33207 (PMC9164099; doi:10.2196/33207)
Supplement: Multimedia Appendix 2 [file games_v10i2e33207_app2.pdf]

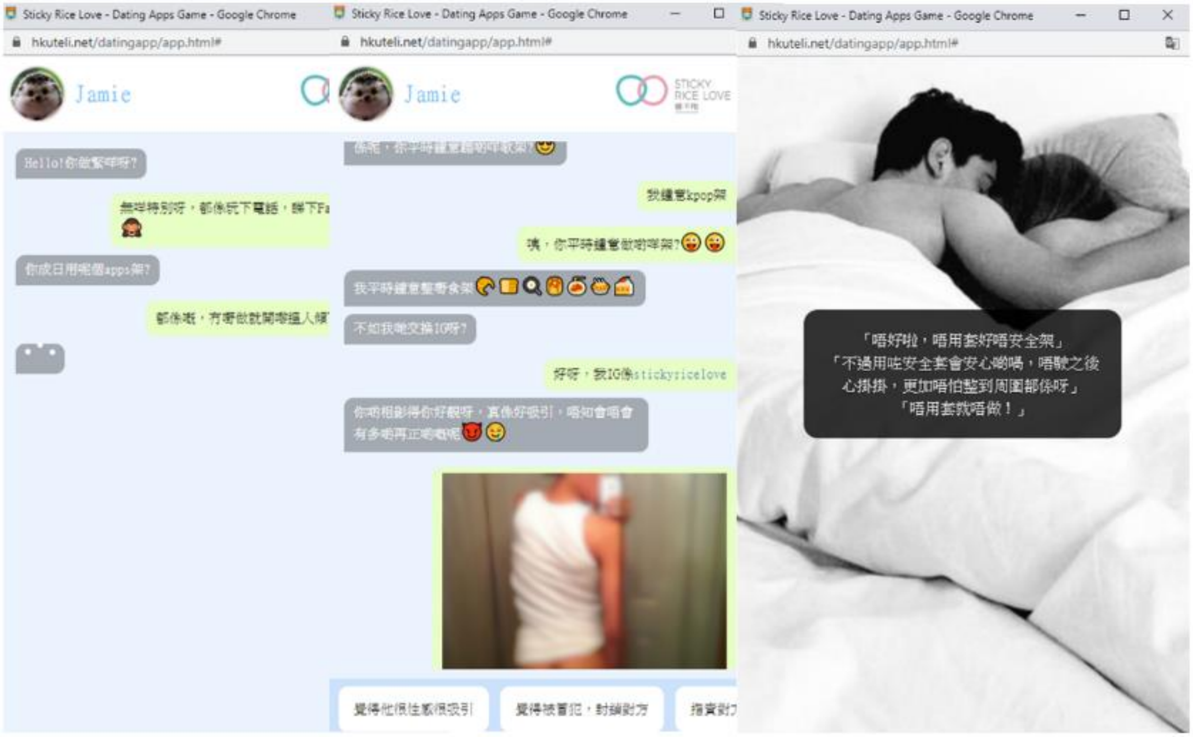

Left:
A: Hello! What are you doing?
B: Nothing special, just using the phone, reading Facebook posts 🙈
A: Do you use this app regularly?
B: Yes, looking for someone to chat with when I'm free

Centre:
A: Btw, what songs do you like? 😎
B: I like K-pop
B: What do you like doing when you are free? 😛😛
A: I like cooking 🥐🍞🍳🥞🍝🍜🍰
A: Shall we add each other on Instagram?
B: Sure, my IG is stickyricelove
A: Your photos are very pretty, really attractive, do you have more photos that are even more stunning? 😈😋
Options:
(Think he is very sexy and attractive) / (feeling offended, block him) / (accuse him)

Right:
Don't be like that, it is not safe not using condoms.
Using condoms make you feel safer, you don't have to worry afterwards (sex), also won't make the place dirty and messy.
If we don't use condoms, then we don't have sex
