# Supplementary material for: Using Video Games to Improve the Sexual Health of Young People Aged 15 to 25 Years: Rapid Review
Source: JMIR Serious Games. 2022 May 19;10(2):e33207. doi: 10.2196/33207 (PMC9164099; doi:10.2196/33207)
Supplement: Multimedia Appendix 3 [file games_v10i2e33207_app3.docx]

Summary of results per intervention^a^.

| Categories | | Decrease in STIs^b^ | Increased contraceptive use | Acquisition of new knowledge regarding sexual health | Change in the perception of risk of pregnancy | Change in the perception of risk of STI | Attitudinal change toward safe sex | Self-efficacy toward sexual health | Decrease in the number of sexual partners | Increase in adherence to prophylaxis or treatment |
| --- | --- | --- | --- | --- | --- | --- | --- | --- | --- | --- |
| **Intervention** | | | | | | | | | | |
|  | The Baby Game | —^c^ | — | Positive | — | — | — | — | — | — |
|  | Romance | — | — | Positive | Positive | — | — | — | — | — |
|  | VODO | — | — | Negative | — | — | Positive | — | — | — |
|  | Choose Your Own Adventure | — | Negative | Positive | Unexpected | Unexpected | Positive | Positive, positive, negative, negative | — | — |
|  | SOLVE^d^ | — | Negative | — | — | — | — | — | — | — |
|  | HIV risk game | — | — | — | — | Positive | — | — | — | — |
|  | BattleViro | — | — | Positive | — | Negative | — | Negative | — | Negative, negative, negative |
|  | Keep it up! | Positive | Positive | — | — | — | — | — | Negative | — |
|  | Viral Combat | — | — | Negative | — | — | — | Negative | Negative | Negative, negative |
|  | MyPEEPS | — | Positive | — | — | Positive | — | Positive | Negative | Negative |
|  | FPSG^e^ | — | — | — | — | — | — | Positive | — | — |
| **Results, n** | | | | | | | | | | |
|  | Positive results^f^ | 1 | 2 | 4 | 1 | 2 | 2 | 4 | 0 | 0 |
|  | Negative results^g^ | 0 | 2 | 2 | 0 | 1 | 0 | 4 | 3 | 6 |
|  | Unexpected results^h^ | 0 | 0 | 0 | 1 | 1 | 0 | 0 | 0 | 0 |

^a^When an intervention used >1 indicator for the same construct, we represented each measure independently.

^b^STI: sexually transmitted infection.

^c^Cells that are empty indicate that the study did not assess that outcome.

^d^SOLVE: Socially Optimized Learning in Virtual Environments.

^e^FPSG: first-person scenario game.

^f^We considered positive results as those in which the treatment produced statistically significantly better results than the control group at a 95% CI.

^g^Negative results are those in which the treatment did not vary significantly from the control group.

^h^Unexpected results are those in which the treatment worked opposite to the expectations. In other words, there were significant differences in favor of the control group.
